# Early responses to H7N9 in southern Mainland China

**DOI:** 10.1186/1471-2334-14-8

**Published:** 2014-01-07

**Authors:** Robin Goodwin, Shaojing Sun

**Affiliations:** 1 School of Social Sciences, Brunel University, Uxbridge, London UB8 3PH, UK; 2 School of Journalism, Fudan University, Shanghai, China

**Keywords:** H7N9, Pandemic influenza, Avian flu, Stigmatisation, Discrimination

## Abstract

**Background:**

H7N9 posed potentially serious health challenges for Chinese society. The previous SARS outbreak in this country was accompanied by contradictory information, while worries about wide-spread influenza led to discrimination worldwide. Early understanding of public threat perceptions is therefore important for effective public health communication and intervention.

**Methods:**

We interviewed 1011 respondents by phone two weeks after the first case. Questions examined risk awareness and media use, beliefs about the emergence of the threat and those most at risk, anxiety about infection and preventive and avoidant behaviours.

**Results:**

Results demonstrate moderate levels of anxiety but relatively high levels of trust towards government officials. Threat emergence was associated with hygiene levels, temperature change, floating pigs in the Huangpu River and migration to the city. Anxiety predicted both recommended and non-recommended behavioural changes.

**Conclusions:**

Comparatively high levels of trust in Chinese government advice about H7N9 contrast positively with previous pandemic communications in China. Anxiety helped drive both recommended and non-recommended behaviours, with potentially important economic and social implications. This included evidence of 'othering’ of those associated with the threat (e.g. migrants). Findings emphasise the need to manage public communications early during new influenza outbreaks.

## Background

Avian influenza A H7N9 was first identified as a novel virus in China on 1^st^ April 2013 [1]. While several variants of highly pathogenic avian influenza have been reported worldwide, poor management and initial denial of SARS in China made the H7H9 outbreak of particular concern [2]. During SARS the Chinese government kept the new outbreak secret, downplaying its emergence and treating it as a local issue best left to local bureaucracy [3]. Communication between provincial localities and bureaucratic departments was poor, and a lack of formal organisation meant poor provision of public health information, partly as a result of a separation between military and public healthcare [3]. Indeed it was not until SARS had spread to several neighbouring countries that it was publicly announced in China [4]. Partially as a result of criticism of their handling of the SARS crisis the Chinese government was keen to rapidly release accurate information about the novel H7N9 threat. Two weeks after the first case the Emergency Office of the Ministry of Health provided a series of behavioural recommendations, including taking greater care when preparing poultry or eggs, increasing hand-washing, ventilating homes and distancing oneself from those seeming to be infected [5]. Further, unlike in previous influenza crises, the Shanghai government closed all live poultry trade markets immediately following the first H7N9 fatality. In this study we examine early public responses to the H7N9 outbreak in southern Mainland China, including lay beliefs about the cause of the outbreak and those most at risk, trust in governmental responses, and uptake of recommended and non-recommended behaviours amongst citizens of Shanghai.

Previous research has indicated that lay beliefs about disease origins and those most at risk, and cultural perceptions about the purchase and preparation of birds, influence disease reporting, individual self-care and avoidance behaviours [6-9]. The coincidence of the new H7N9 threat and the wide-spread reporting on the social media site Weibo of thousands of diseased pigs floating in a river key to Shanghai’s water supply led to a municipal government press release denying any link [10]. Risk associations between avian influenza and the poultry industry echoed both official communications from the Chinese government and broader public concerns about farming methods during earlier influenza A outbreaks [7]. In particular, poor ventilation, unsanitary practices at markets and by chicken sellers [7], and the transport of poultry on long distance roads [11], was associated with a previous outbreak of H5N1. During both SARS [12] and previous influenza outbreaks [13] association of the viruses with particular 'others’ allowed individuals to feel relatively safe from infection, and contributed to the victimisation of some groups [14]. In particular, newly arrived migrants were seen as bringing new threats to an indigenous population [15]. Other causal attributions – in both public discourse and the scientific literature – have linked influenza outbreaks to temperature and climatic factors [16]. In this paper we consider associations between H7N9 and 'floating pigs’, new migrants, the poultry trade and wider temperature and climactic change.

Previous work has also stressed the significance of source credibility in the context of risk and health communication [17]. In recent years, the credibility of Chinese government pronouncements has been increasingly questioned [18]. In this paper we consider sources of credibility in announcements about H7N9, comparing Chinese government representatives (from relevant government offices), with “Chinese experts” (scholars or commentators on public health) and “Western experts” (scholars or leaders in public health from the World Health Organisation or Western institutions). Beliefs about infection risks may change rapidly during an epidemic [9], making early understanding of cultural and psychological risk factors critical for subsequent health communication. We therefore report beliefs and understandings of this novel pathogen in southern Mainland China two weeks after its emergence, and associations with preventive behaviour.

## Methods

Ethical approval was received from the relevant IRB board (Fudan Research Committee, School of Journalism) at Fudan University. We interviewed 1011 participants in the Shanghai region between April 12 and April 20th 2013 (599 female, 412 male; *M* age 49.5 (SD 16.8)), using random digit dialing of home phones. Total H7N9 confirmed laboratory cases/fatalities in Mainland China during this time ranged from 43/11 (April 12th) to 96/18 [1]. Data were collected via random digit dialing and computer-assisted telephone interviewing. Inclusion criteria were participants aged 18 or above who had heard of H7N9. Random phone numbers were generated by a computer system, developed by the Nankang IT Company specializing in survey and data collection. All interviewers were required to attend a 40-minute training session before using the system. When placing a call, the interviewer identified him/herself as calling from the university survey center, and explained to interviewees the purpose, procedure and confidentiality issues. Interviewees were informed that their participation was fully voluntary and they could end the interview at any time. Another household member was solicited for participation if an interviewee did not meet the age criteria or refused to participate, but only one household member was permitted to take the survey. Each generated phone number was tried three times before being replaced by an alternative random number. Three thousand and fifty-four telephone numbers were dialled, of which 1082 gave interviews, and 1972 refused to participate (response rate 35%). A further 32 gave incomplete responses (16 as a result of technical problems during the interview), 39 were deleted as they had not heard of H7N9 or were out of the age quota group, leaving an eligible 1011 participants. Response rates compare positively to analogous public surveys on swine flu [19]. Table 1 breaks down participants by age, sex and occupation, comparing these categories with wider Shanghai data [20].

**Table 1:** Sample characteristics

**Variable**	**Sample characteristic (N = 1011)**	**Population profile**[20]
Sex (female)(male)	59%, 41%	49%, 51%
Age groups		
-18-30	18.3%	29.9%^a^
-31-46	26.3%	27.2%
-47-64	34.7%	25.2%
-65 and above	20.7%	10.1%
Occupation		
- Professional technicians or experts	13.1%	12.8%
- Government employees	2.6%	3.4%
- Business	20.1%	22.4%
- Service industry	17.1%	11.8%
- Agriculture, fishery, and farming	6.7%	11.3%
- Factory worker	37.5%	38.2%
- Other	2.9%	0.1%

^a^NB: Population statistics include those aged under 18; all our respondents were 18 or over.

Interviewers asked five sets of questions (Table 2), based on previous work on risk representations [19], risk communications [13], and preventive and avoidant behaviours during epidemic/pandemic threat [9,19,21]. Questions covered 1) awareness of the threat 2) use of media, and perceived credibility of communicators 3) perceived reasons for the emergence of the threat 4) those perceived to be at greater risk of infection on the basis of previous research on H1N1 (elderly people, children, young people, migrants, those with weakened immunity) 5) anxiety about infection and 6) behavioural responses to the threat.

**Table 2:** Survey items and response categories

**Topic**	**Question**	**Possible responses**
Awareness	Have you previously heard of H7N9	Yes, no
	Did you use the following media to learn about H7N9? TV, Radio, Newspapers, Micro-blogs, Social networks, Cell phone news	Yes, no (for each item)
Credibility	To what extent do you believe the following information sources about H7N9? The Chinese government; Chinese domestic experts; Western experts	Do not believe, moderately believe, strongly believe (for each item)
Associations with H7N9	To what extent do you associate each of the following with H7N9? Floating pigs in the Huangpu; New migrants to Shanghai; Poor Chinese hygiene in general; The unhygienic poultry trade; Recent temperature fluctuations; Global climate change	Related, unrelated, uncertain (for each item)
Groups at risk of H7N9	What is the risk of the following groups being at risk from H7N9? Those with regular contact with poultry; Migrants; Children; Young people; Elderly people; Those with weak immunity	More at risk than me, similar risk than me, less risk than me, unsure (for each item)
Anxiety about H7N9	Do you worry you (your family) will be infected with H7N9?	Do not worry, worry a little, worry quite a lot, worry a great deal
	Since the H7N9 outbreak, have you been thinking more about life and death	No, a little more, a great deal more
	Did H7N9 bring any difficulty to your daily life?	None, just a little, some, a lot
	What is your personal perceived probability of infection	Unlikely, a little unlikely, very likely
Behavioural responses to H7N9	Following H7N9 did you: Buy Chinese medicine to prevent the virus; Buy western medicine to prevent the virus; Increase house ventilation; Avoid buying poultry or eggs; Change cooking methods; Avoid crowded places; Avoid recent migrants to Shanghai; Decrease public transport use; Postpone or cancel travelling; Change washing habits	Yes, no (for each item)

### Data analyses

Analyses were conducted using SPSS 18.0. We report frequencies of beliefs about H7N9 and those most at risk and behavioural responses to the threat. Personal worry and worry about infection in the family were highly related (Pearson correlation (r) = .79) and combined as 'Anxiety’. Outcome variables were divided into behaviours recommended by the Chinese Ministry of Health [5] and avoidance behaviours [19], plus purchase of medicines. Differences in trust in communications were examined in a univariate ANOVA within-subjects General Linear Models (GLM); relationships between anxiety, causal associations with H7N9 and risk group perceptions through Spearman correlations. Binary logistic regressions examined multivariate associations between anxiety and media usage, risk perceptions and outcome behaviours, adjusting for age and sex. To minimize Type 1 errors all reported significance levels are at .01 or below.

## Results

Respondents most associated H7N9 with poor hygiene or climatic changes (Table 3). At least half our respondents rated those in greater contact with poultry, with weakened immunity, elderly people or children as at enhanced risk; 38% suggested migrants were at greater risk. Television was the most frequent source of information about H7N9 (mentioned by 879 (87%) of respondents). Overall 5% of respondents relied solely on social media (microblogs, social media or cell phone newsletters), 28% combined media sources. When communicating about the threat Chinese officials or experts were believed more than Western experts (*F* (2, 1430) = 142.5, *p* = .001).

**Table 3:** Beliefs about causation, perceived credibility of information sources and risk perceptions following a H7N9 outbreak

**Item**				
**Beliefs about causation**		***Related*** **(N, %)**	***Uncertain***	***Not related***
	Floating pigs	349 (34.5)	272 (26.9)	385 (38.0)
	New migrants	232 (23.1)	246 (24.5)	525 (52.3)
	Poor Chinese hygiene	622 (61.7)	272 (27.0)	114 (11.3)
	Unhygienic poultry trade	784 (77.6)	90 (8.9)	136 (13.5)
	Recent temperature fluctuations	548 (54.4)	95 (9.4)	364 (36.1)
	Global climate change	450 (44.6)	202 (20.0)	356 (35.3)
**Trust in communications**		***Strongly believe***	***Moderately believe***	***Do not believe***
	Chinese government	360 (36.5)	529 (53.6)	98 (9.9)
	Chinese experts	191 (20.0)	525 (54.9)	241 (25.2)
	Western experts	87 (11.8)	339 (45.9)	312 (42.3)
**Beliefs about relative risk**		***More at risk than me***	***Similar or less risk***	***Unsure***
	Regular contact with poultry	863 (85.4)	60 (5.9)	88 (8.7)
	Migrants	381 (37.7)	420 (40.6)	210 (20.8)
	Children	522 (51.6)	383 (37.9)	106 (10.5)
	Young people	59 (5.8)	821 (81.2)	131 (13.0)
	Elderly people	736 (72.8)	198 (19.5)	77 (7.6)
	Those with weak immunity	815 (80.6)	106 (10.5)	90 (8.9)

Respondents demonstrated low to moderate anxiety about H7N9 (Mean = 1.7/4). Forty-one respondents (4%) were 'worried a great deal’ about personal infection, 121 (12%) worried 'quite a lot’, 264 (26%) worried a little and 582 (58%) not worried. Regarding concern about their family being infected 83 (8%) were 'worried a great deal’, 169 worried 'quite a lot’, 260 (26%) worried a little and 498 (49%) not worried. Sixty-seven percent (642) thought it unlikely that they personally would be infected, 310 (31%) that this was 'a little likely’ and 11 (1%) that this was very likely. Since the H7N9 outbreak, 97 (10%) had been thinking a great deal more about life and death, 400 (40%) a little more about this and 500 (50%) no more than usual. Finally, 42 (4%) reported that H7N9 had brought a lot of difficulty to their daily life, 284 (28%) some difficulty, 120 (12%) a little difficulty and 562 (56%) no difficulty.

Table 4 gives associations between anxiety and infection beliefs. Participants with greater anxiety associated floating pigs and the poultry trade hygiene with the outbreak, and thought children and the elderly were at greater risk. These associations, while significant, were however generally weak. The media source of information about H7N9 was unrelated to anxiety (for only traditional media odds ratio (OR) = .90, 95% confidence interval (CI) .76-1.06 *p* = .20; for only social media OR = 1.21, 95% CI .87-1.68, *p* = .26)

**Table 4:** Anxiety, associations with infection and perceptions of risk group

**Associations with H7N9**	**Correlation Spearman r with Anxiety (number)**
- Floating pigs	.18** (1004)
- New migrants	.07 (1001)
- Poor Chinese hygiene	.05 (1006)
- Unhygienic poultry trade	.11** (1008)
- Recent temperature fluctuations	.07 (1006)
- Global climate change	.05 (1007)
Risk group	
- Regular contact with poultry	.06 (928)
- Migrants	.01 (800)
- Children	.14** (904)
- Young people	.08 (879)
- Elderly people	.10* (933)
- Those with weak immunity	.08 (920)

*p < .01; ** p < .001.

Note numbers in risk group exclude those who were uncertain.

Table 5 provides recommended and non-recommended behavioural responses to H7N9. The most widely adopted recommended response was home ventilation, with half reporting changing cooking behaviours, half avoiding sick people, nearly half changing washing habits. The most frequent non-recommended changes were the avoidance of buying poultry or eggs, and the avoidance of crowds. A minority of respondents reported mask wearing, cancelling or changing public transport travel plans, avoiding being physically close to migrants, or purchasing Chinese or Western medicine.

**Table 5:** Behavioural responses to H7N9

**Items**	**No. (%) positive responses**
*Behaviours recommended by the Chinese Ministry of Health*[5]	
Changes in cooking behaviour (poultry/eggs)	515 (51.5)
Change hand washing habits	431 (42.8)
Ventilation at home	675 (66.9)
Avoiding those who are sick	515 (51.8)
*Non-recommended behaviours*	
Avoided buying poultry/eggs	711 (70.5)
Avoiding public transport	128 (12.8)
Avoiding crowds	581 (57.8)
Avoiding recent migrants entering Shanghai	169 (17.1)
Wearing a mask	301 (29.7)
Cancelling travel plans	234 (23.3)
*Additional purchases*	
Purchasing Chinese medicine	98 (9.7)
Purchasing Western medicine	30 (3.0)

Table 6 provides predictors of recommended and non-recommended behaviours. As can be seen, participants carrying out two or more recommended behaviours were significantly higher on anxiety, perceived probability of personal infection and mortality awareness. Changing behaviours per the recommendations was associated with attributing H7N9 to floating pigs, hygiene standards in the poultry industry, hygiene in Chinese society, and migration and by perceiving children and those with weak immunity as particularly at risk. Participants who took part in non-recommended behaviours were also higher on anxiety, perceived probability of infection and mortality awareness. Non-recommended behavior was predicted by associations of H7N9 with general hygiene standards and viewing children and those with weak immunity as at enhanced risk. Finally, purchasing Chinese medicine was associated with anxiety (OR = 1.64, 95% CI 1.31-2.04, *p* < .001) and death awareness (OR = 1.51, 95% CI 1.11-2.07, *p* = .01). The purchase of Western medicine was also associated with anxiety (OR = 1.64, CI 1.13-2.37 *p* = .01) and death awareness (OR = 2.12, 95% CI 1.24-3.64 *p* = .01).

**Table 6:** Predictors of recommended and non-recommended behaviours

**a. Contrasting those who performed two or more recommended behaviours (N = 650) with those who did not (N = 328)**		
**Variable**	**Odds ratio, significance**	**95% confidence interval**
Anxiety	1.73**	1.45-2.08
Perceived probability of infection	1.77**	1.31-2.38
Mortality awareness	2.22*	1.76-2.81
Associations with H7N9		
- Floating pigs	1.50**	1,27-1.77
- New migrants	1.31*	1.10-1,55
- Poor Chinese hygiene	1.30**	1.12-1.52
- Unhygienic poultry trade	1.35*	1.12-1.63
- Recent temperature fluctuations	1.07	.93-1.24
- Global climate change	1.06	.91-1.23
Risk group		
- Regular contact with poultry	.99	.63-1.61
- Migrants	1.14	.92-1.41
- Children	1.35**	1.12-1.62
- Young people	1.00	.79-1.27
- Elderly people	1.31	1.03-1.66
- Those with weak immunity	1.77**	1.29-2.41
**b. Contrasting those who reported two or more avoidance behaviours (N = 838) versus those who did one or none (N = 127)**		
**Variable**	**Odds ratio**	**95% confidence interval, significance**
Anxiety	1.52*	1.17-1.98
Perceived probability of (personal) infection	2.07*	1.30-3.32
Mortality awareness	1.84**	1.31-2.57
Associations with H7N9		
- Floating pigs	1.24	.99-1.57
- New migrants	1.07	.85-1.37
- Poor Chinese hygiene	1.54**	1.25-1.90
- Unhygienic poultry trade	1.14	.88-1.47
- Recent temperature fluctuations	1.09	.89-1.33
- Global climate change	1.16	.94-1.44
Risk group		
- Regular contact with poultry	1.69	.99-2.88
- Migrants	1.22	.91-1.64
- Children	1.41*	1.09-1.81
- Young people	1.15	.81-1.62
- Elderly people	.92	.65-1.32
- Those with weak immunity	1.77*	1.21-2.60

*Note* For a) OR > 1 indicates more likely to report 2+ recommended behaviours; for b) OR > 1 indicates more likely to report 2+ non-recommended behaviours. All analyses control for age and sex.

*p < .01; ** p < .001.

## Discussion

Large populations and high population density make it critical to understand public responses to novel influenza viruses in China. Mixed messages from official sources can produce confusion, permitting anxiety to spread rapidly within families and communities, and reducing important behavioural change [2]. In this study relatively high levels of trust in Chinese government advice about H7N9 contrast positively with previous pandemic communications in this country. This may be the result of a new determination to provide rapid, reliable information on such threats in this country [22]. National and regional media, along with Chinese CDC, disseminated preventive knowledge and local governments updated their pandemic status on a daily basis. Mainstream media aside, the government made heavy use of new media, particularly Chinese microblogs to update information rapidly while cell phone news was updated regularly [23]. At the same time there was little evidence of the social amplification of anxiety via social media previously observed in the literature [24]. This may be a consequence of the fear of detention that followed arrests for spreading online rumours about the outbreak.

Respondents associated temperature changes, the poultry trade and unexplained local events (e.g. floating pigs) with H7N9. Associations with seasonal and climatic variations in influenza, also suggested in the research literature [25], imply high awareness of temporal factors. Anxiety levels, while relatively low, were higher than those reported early on during H1N1 in Mainland China, where 72% claimed they were 'not worried’ about infection [6]. Consistent with studies of pandemic threat elsewhere anxiety helped drive both recommended and non-recommended behaviours [19]. Almost a quarter of respondents reported cancelling travel plans, emphasising the significant economic impacts of pandemic threat [2]. There was also some evidence of stigmatisation and the 'othering’ of those associated with the threat (e.g. migrants). Such findings have also been reported elsewhere during pandemic threat [13,21]. However, viewing children or those with weak immunity as at risk was also associated with reports in changes of behaviour, suggesting some sense of communal responsibility for some vulnerable groups.

The present study had some limitations. The telephone interview format employed meant we could not contact those without home phones and limited the length of the survey. As those without home phones are more likely to be temporary migrants within the city we were consequently less able to draw from a migrant population for our study. High levels of trust towards the government may reflect the relatively large number of respondents in our sample aged 65 or over. Data was from self-reports and therefore behaviour cannot be independently verified (e.g. hand washing behaviours). The need for immediate data collection reduced sample size, while random dialling potentially limited response rate.

## Conclusions

At the time of writing there has been little evidence of human-to-human transmission of H7N9. Nevertheless our findings have important implications for clinicians and those working in health communication. Local expertise was particularly valued by our respondents, suggesting the efficacy of a new government strategy to provide rapid, transparent information. Other national authorities elsewhere may learn from this example when faced with emergent influenza threats. At the same time, even at this early stage of the pandemic threat, respondents reported buying preventive medicines, particularly local products. Public officials need to be aware that increased public anxiety may rapidly produce medical shortages. 'Common sense’, lay beliefs about those at risk, and appropriate behaviours to adopt to avoid infection influence adherence and self-care, but are rarely considered by practitioners. Perceptions of enhanced risk from some groups, such as those from outside a city, may cause socially disruptive behaviours, particularly if threat levels increase. This emphasises the continuing need for clear risk communications in order to abate potential discrimination and public disorder during influenza threat.

## Competing interest

All authors report no competing interest disclosures.

## Authors’ contributions

Study concept and design: RG, SS. Acquisition of data: SS. Analysis and interpretation of data: SS, RG, Drafting of the manuscript: RG, SS. Statistical analysis: RG, SS. Critical revision of the manuscript for important intellectual content: RG, SS. Final approval of manuscript: RG, SS. Obtained funding: SS. Administrative, technical, or material support: SS. Study supervision: SS. SS had full access to all the data in the study and takes responsibility for the integrity of the data and the accuracy of the data analysis.

## Pre-publication history

The pre-publication history for this paper can be accessed here:

http://www.biomedcentral.com/1471-2334/14/8/prepub
